# Rural–urban inequalities in health care utilization in Bhutan: a decomposition analysis

**DOI:** 10.1186/s12939-024-02178-4

**Published:** 2024-04-12

**Authors:** Jayendra Sharma, Milena Pavlova, Wim Groot

**Affiliations:** 1 Department of Health Services Research, CAPHRI, Maastricht University Medical Center, Faculty of Health, Medicine and Life Sciences, Maastricht University, P.O. Box 616, Maastricht, 6200 MD the Netherlands; 2 Thimphu, Bhutan

**Keywords:** Bhutan, Health care utilization, Health inequality, Decomposition analysis, Universal health coverage

## Abstract

**Background and objective:**

On the trajectory towards universal health coverage in Bhutan, health equity requires policy attention as significant disparities exist between urban and rural health outcomes. This paper examines health services utilization patterns, inequalities and their socio-economic determinants in rural and urban areas and decomposes the factors behind these differences.

**Methods:**

We used the Bhutan Living Standard Survey 2017 to profile health services utilization patterns and equalities. We employed two different decomposition analyses: decomposition of mean differences in utilization using the Oaxaca–Blinder decomposition framework and differences in the income-related distribution in utilization using recentered influence function regressions between rural and urban areas.

**Results:**

Significant differences exist in the type of outpatient services used by the rural and urban population groups, with those living in rural areas having 3.4 times higher odds of using primary health centers compared to outpatient hospital care. We find that the use of primary health care is pro-poor and that outpatient hospital resources is concentrated among the more affluent section of the population, with this observed inequality consistent across settings but more severe in rural areas. The rural–urban gap in utilization is primarily driven by income and residence in the eastern region, while income-related inequality in utilization is influenced, aside from income, by residence in the central region, household size, and marriage and employment status of the household head. We do not find evidence of significant mean differences in overall utilization or inequality in utilization of inpatient health care services.

**Conclusions:**

While the differences in average contacts with health services are insignificant, there are prominent differences in the level of services availed and the associated inequality among rural and urban settings in Bhutan. Besides, while there are obvious overlaps, factors influencing income-related inequality are not necessarily the same as those driving the utilization gaps. Cognizance of these differences may lead to better informed, targeted, and potentially more effective future research and policies for universal health coverage.

## Introduction

Access to necessary care is a major determinant of population health and a key tenet of progress toward universal health coverage (UHC) [1, 2]. Equitable access is firmly grounded in the core principles of UHC, which advocates that health care must be distributed according to need and regardless of the ability to pay. Understanding the magnitude and determinants of access and inequalities in health and health care is generally important for health policy decisions. Empirical evidence in this area is vital for targeted approaches toward vulnerable population groups.

Research on inequality in health care utilization has received substantial attention. It is important to highlight the difference between access and utilization of health services, which are often used interchangeably in policies, but are distinct terminologies subject to different interpretations [3–5]. Broadly, access measures individuals’ ability to navigate within the health care delivery ecosystem, while utilization measures actual encounters of care, often determined by individual attributes as well as socio-economic and organizational factors [3].

Measuring utilization and inequalities is, however, just one part of the answer. We must also explain why inequalities exist and which factors fuel these observed inequalities. Such evidence would enable policies to target those underlying dimensions and factors better. Two streams of analyses are prominent in explaining socio-economic inequalities in health and health care; decomposing group differences in the mean outcome of interest (for example, mean utilization of health care) and decomposing differences in the distribution of the outcome of interest across socio-economic status (usually, decomposition of the concentration index). Absolute or relative measures used in isolation do not fully convey inequality and should be reported together with utilization levels [6, 7]. A low level of inequality is not, in itself, a sufficient reason to aim for UHC. For instance, low inequalities in the context of low utilization levels across all income groups signal poor UHC status. Therefore, inequality measures must be considered together with overall service use [6, 7]. Inequality differences could be caused either by changes in the difference in the means or the distributions, and separating these effects makes the inequality changes more transparent [8]. By addressing both the mean effect (first approach) and the distribution effect (second approach), our understanding of the service use, patterns and the extent and drivers of inequality is much enhanced.

This paper focuses on health care use and related inequalities in Bhutan. Bhutan’s developing economy, primarily driven by public sector investments, has grown steadily in the last 60 years. Between 2000 and 2017, Bhutan’s per capita GDP increased more than four times and currently stands at US$3,100 [9]. The country has maintained a strong track record in reducing poverty, particularly in the recent decade. The share of the population living below the national poverty line has been significantly reduced between 2007 and 2017, from 23.2% to 8.2%, with the national poverty line of Ngultrum 2195.95 (approximately USD 33^1^) per person per month in 2017 [10]. Regional and geographical disparities, however, persist and poverty in rural areas (11.9%) is still considerably higher than in urban areas (0.8%) [10]. The challenge of inequality in Bhutan is manifested in the Gini index of consumption, which was estimated at 0.38 in 2017, providing for, on average, a person in the top 20% of the population consuming 6.7 times more resources than a person in the bottom 20% of the population [10].

These disparities translate into differences in health service utilization and outcomes, particularly between the rural and urban areas of the country. According to the Bhutan Health in Transition review [11], health equity requires policy attention with disparities existing in access to and utilization of health services as well as in health outcomes between urban and rural areas. Currently, 62.2% of Bhutan’s population resides in the country’s rural areas [12], characterized by scattered settlements, difficulties in access to services and relatively poorer socio-economic conditions. In terms of health outcomes, the under-five mortality rate was 2.75 times higher in rural than in urban Bhutan [13]. Neonatal mortality, which contributes to 70% of infant deaths in Bhutan, was 86.2% more in rural areas compared to urban areas [14]. Similarly, about twice as many children are stunted and underweight in rural areas compared to urban areas [15]. In terms of access and utilization of health services, a considerable amount of literature suggests differences among people by their urban or rural residence [11, 16, 17]. A prominent challenge that intersects with health service access and utilization patterns in Bhutan is the issue of patients bypassing primary health care to access higher levels of care. According to one study, more than 50% of patients with five minor illnesses, that could be handled at the primary care level, were treated at secondary or tertiary hospitals [16]. Besides the absence of a robust gatekeeping function [11], a complex interplay of demand and supply side factors appears to influence bypassing primary health care facilities [18].

Like many other countries, Bhutan has prioritized health equity as a critical pillar of its UHC goals [19]. Besides maintaining the health system as predominantly public and universal in scope, there are several programs and initiatives such as mobile clinics to take specialized health services closer to the far-flung and remote areas of the country, people-centered care initiative, and the ongoing review of the national health policy, particularly to address geographical constraints and challenges of service delivery in the far-flung and rural areas of the country. Yet, it is essential to understand the factors driving these gaps in utilization and observed inequalities, which must be adequately examined, to inform more effective and targeted policy and programmatic interventions.

The objectives of this study are (i) to assess rural–urban differences in the utilization of health services in Bhutan, (ii) to examine the determinants of health services utilization, (iii) to assess socio-economic related inequalities in health services utilization, and (iii) to analyze the contribution of these determinants to rural–urban inequalities in the utilization of health services. While this study aims to contribute to UHC policy and programmatic actions in Bhutan, it offers a conceptual design and scope for comparative analysis among low- and middle-income countries facing similar constraints and issues in their progress towards UHC. In particular, it contributes to a more nuanced and transparent inequality monitoring through a comparison of group differences in the mean outcome of interest and the distribution of the outcome of interest across socio-economic status.

## Methods

### Data source and variables construction

We use secondary data from the 2017 wave of the Bhutan Living Standard Survey (BLSS). The BLSS questionnaire covers various aspects of living standards and social well-being, including demography, health, education, housing, household income and expenditures, and use and satisfaction with public services. The BLSS 2017 was designed to cover all twenty districts, both urban and rural areas, and four major townships. A stratified two-stage sampling design was adopted with *Chiwogs*/villages (rural) and enumeration areas (urban) adopted as primary sampling units, while households formed the secondary sampling units. Institutional households such as residential schools/colleges and monastic centers were excluded from the sampling frame. Trained enumerators visited the sampled households and collected data. Three failed attempts to make contact with household members were counted as non-response, and of the 11,812 households planned, 11,660 participated, canvassing 48,639 individuals, with a response rate of 98.7%. Additional details of the survey methodology and updates in the questionnaire during these surveys are available in the BLSS 2017 report [20].

The outcome variables of interest cover the usage spectrum of health services, classified into three areas: used outpatient care in case of ill health or injury in the past month; type of outpatient care used when experienced ill health or injury in the past month; and, used inpatient hospital care in the past year. These dichotomous variables were derived and constructed from the survey, calculated separately considering the differing inclusion and recall periods, as follows:

- For the use of outpatient health services: We included in this variable those who reported experiencing ill health or injury in the preceding month from the survey question “Did [NAME] suffer from sickness or injury in the last four weeks?”. A subsequent question, “Did [NAME] visit/consult a health provider without staying overnight in the health facility (referral hospital/hospital/Basic Health Units”, with answers coded as yes = 1 and no = 0, was used to determine the need-based utilization of outpatient health services in the past one month. This resulted in a sample of 5904 individuals.
- For the type of outpatient care used: We extracted from the sample only those that have used outpatient care in hospitals or primary health care centers. Since the survey question allowed respondents to score multiple visits, responses for the “first visit” were used to distinguish between hospital and primary health care centers. 76.8% of all responses were for the first visit, and the first visit covered all respondents. A total of 4021 individuals qualified for inclusion in the sample. We use the term outpatient care to refer to care provided at outpatient hospital facilities and primary health centers.
- For the use of inpatient health services: We derived this variable from the question “Was [NAME] admitted to staying overnight at a medical facility (referral hospital/hospital/BHU) in the last 12 months?” with answers coded as yes = 1 and no = 0. Inpatient care is purely hospital-based care. The recall period was maintained at one year. Utilization of hospital services for maternity/delivery care has been excluded from the analysis. The entire sample, or a total of 48,639 individuals, was considered for the analysis.

We used a set of explanatory variables, with both positive and negative impacts reported on the outcome variables, informed by existing and relevant literature in Bhutan [11, 16, 17] and elsewhere [21–25]. Sex was defined as male or female. Age was categorized into three groups: less than 30, 30–59, and 60 years and older. Any functional limitations/disability (seeing, hearing, walking, remembering, or self-care) was defined as yes/no. Geographical and socio-economic predictors included geographical region (three nominal categories), urban/rural area, education (three ordinal categories), marital status (three nominal categories), employment status (yes/no), household size (three ordinal categories) and health facilities within 1-h distance (yes/no). We used the per capita household consumption expenditure to rank the population and classify individuals into quintiles.

### Statistical analysis

#### Health care utilization

We began with a descriptive analysis of the utilization patterns for different categories of services. We analyzed each variable independently. The variable on the use of outpatient care is constructed as the use of care when ill or injured in the past one month. The variable on use of inpatient care is constructed to assess utilization patterns over one year. We used logistics regression (three models representing rural, urban, and total) to examine factors associated with the usage of these services.

#### Decomposition of the mean effect

We proceeded with our decomposition analysis investigating the factors behind the mean differences in utilization between rural and urban areas (mean effect). The analysis quantifies the contribution of individual factors to gaps in the mean utilization of health services between rural and urban areas. We employed the Oaxaca–Blinder decomposition framework [26, 27]. This framework allows the decomposition of outcome variables between two groups into two parts: differences in observed characteristics (explained component/characteristics effects) and those attributable to differences in estimated coefficients (coefficient effects).

The Oaxaca-Blinder decomposition method has traditionally been based on a linear regression framework requiring coefficient estimates from the linear regression and sample means of the explanatory variables. When the outcomes of interest are binary, the standard linear method yields inconsistent parameter estimates and misleads decomposition results [28]. We adopted the Fairlie decomposition approach, which is an extension of the Blinder-Oaxaca decomposition technique to binary outcome measures [29, 30].

Following Fairlie [29], the average difference in the utilization of health services among rural and urban areas can be expressed as:

1 $${\overline{Y} }^{r} - {\overline{Y} }^{u} = \left[{\sum }_{i =1}^{{N}^{r}}\frac{F \left({X}_{i}^{r} {\beta }^{r}\right)}{{N}^{r}} - {\sum }_{i =1}^{{N}^{u}}\frac{F \left({X}_{i}^{u} {\beta }^{r}\right)}{{N}^{u}}\right] + \left[{\sum }_{i =1}^{{N}^{u}}\frac{F \left({X}_{i}^{u} {\beta }^{r}\right)}{{N}^{u}} - {\sum }_{i =1}^{{N}^{u}}\frac{F \left({X}_{i}^{u} {\beta }^{u}\right)}{{N}^{u}}\right]$$

Where ${\overline{Y} }^{r}$ and ${\overline{Y} }^{u}$ are the average probabilities of use of health services in rural (r) and urban (u) areas respectively. ${X}_{i}$ is the mean vector of independent variables in the respective groups (rural and urban) and $\beta$ is the vector of coefficients estimated separately for each of the two groups. *N* stands for the number of observations in respective groups, and *F* is the cumulative logistic distribution function. The first bracketed term represents the explained component or endowment effects which is the proportion of the difference between rural and urban areas in the utilization of health services explained by differences in the distribution of characteristics. The second bracketed term represents the coefficient effects, which estimate the difference between rural and urban areas due to differences in unmeasurable or unobserved determinants. This is the portion of the gap that may be due to wealth discrimination, differences in the availability of health services, differences in attitudes between wealth groups, or other unmeasured characteristics [29]. We used the *fairlie* command which performs nonlinear decomposition of binary outcome differentials [31] in Stata 17 [32]. The effects of categorical socio-economic variables are modelled by including dummy variables (assigned 0 and 1) for the different categories in the regression equation, with the base category omitted to avoid collinearity. Detailed decomposition has been estimated based on 1000 replications of one-to-one matching of cases between the two groups. We set the reference coefficients from the model having a higher probability of utilization.

#### Concentration index

To estimate the income-related inequality in the use of different health services, we measured inequality by concentration curves and the associated concentration indices. The concentration curve plots the cumulative percentage of use of various health services on the vertical axis against the cumulative percentage of people ranked by their socio-economic status on the horizontal axis, from poorest to the richest. The concentration index (CI) is calculated as twice the area between the concentration curve and the 45° line of equality, as in the following equation [33]:

2 $$CI = \frac{2}{\mu } cov \left(h, r\right)$$

Where *h* is the utilization variable, $\mu$ is its mean, *r* is the fractional rank of individuals in the living standard distribution and *cov* is the covariance between the health variable and the fractional rank of the stratifying variable. Considering the binary outcome variable, Erreygers’ normalization/correction to the CI was used [34, 35]. The value of CI varies between -1 and + 1, with the sign of the CI indicating the direction of the relationship and its magnitude reflecting both the strength of the relationship and the degree of variability in the health care variable.

#### Decomposition of the distribution effect

Finally, we decomposed the Erreygers’ corrected concentration index to identify factors related to differences in income-related distribution in utilization or identify variables which have a significant influence on this index, between rural and urban areas (distribution effect). We employed the extension to the Oaxaca–Blinder decomposition that relies on recentered influence function (RIF) regressions as suggested by Firpo et al. [36, 37]. This method allows for general distributional measures to be decomposed in the same way as means can be decomposed using the general Oaxaca–Blinder method [37]. The main advantage of using the RIF-regression method in an Oaxaca-Blinder type of decomposition is that it provides a linear approximation of highly nonlinear functionals [37], and is able to apply to all forms of inequality measures [38]. This two-stage method involves dividing the distributional changes into endowment and composition effects by estimating a logit model (stage 1) and dividing the components into the contribution of each individual covariate using the RIF regression (stage 2), the detailed mathematical derivation of which is available in the study by Firpo [37].

The decomposition was undertaken using the *oaxaca_rif* command using the *eindex* option (using Erreygers’ index as the distributional statistic) [39]. The observed differences in the concentration of use of health services were divided into two components: attributable to endowment differences between rural and urban areas or explained components and differences in coefficients or unexplained components. Dummy variables were used to model the effects of categorical variables in the regression equation, with the base category omitted to avoid collinearity. As suggested by Firpo et al. [36], we estimated standard errors through the bootstrap method and applied 100 replications.

## Results

### Descriptive analysis

Of the 48,639 individuals enumerated during the survey, 5,989 reported being ill or injured in the month preceding the survey. Of those who reported ill health or injury, 1,071 individuals used services at the primary health centers, 2930 individuals used hospital outpatient services, and 1,883 individuals did not use health care. In addition, 85 individuals used health services, the categories of which were unspecified in the survey or were availed from informal practitioners or private providers. During the preceding year, 1,496 individuals were admitted for inpatient care in hospitals. Table 1 presents the distribution of health services utilization and the population groups’ socio-economic characteristics.

**Table 1:** Utilization of health services and socio-economic characteristics

	**Experienced ill health or injury in the past month**	**Utilized outpatient health services when experienced ill health or injury in the past month**				**Utilized hospital inpatient services in the past year**
		**Primary health care facilities**	**Hospital outpatient services**	**Other services**^a^	**Did not use outpatient care**	
	*N* = 5989	*N* = 1091	*N* = 2930	*N* = 85	*N* = 1883	*N* = 1496
**Socio-economic characteristics**	%	%	%	%	%	%
Urban	44.40	18.61	54.88	69.41	41.90	39.37
Rural	55.60	81.39	45.12	30.59	58.10	60.63
Male	41.78	37.86	41.71	51.76	43.71	41.71
Female	58.22	62.14	58.29	48.24	56.29	58.29
Age less than 30 years	43.96	41.52	44.85	45.88	43.92	46.26
Age 31–60 years	39.42	40.15	38.60	43.53	40.10	37.57
Age above 60 years	16.61	18.33	16.55	10.59	15.99	16.18
Never married	35.57	35.29	36.08	38.82	34.78	29.28
Married or together	55.03	54.17	55.46	55.29	54.86	60.96
Divorced or separated	9.40	10.54	8.46	5.88	10.36	9.76
No formal education	57.02	67.19	54.54	44.71	55.55	58.16
Primary, secondary and vocational	39.87	31.07	42.29	50.59	40.73	38.37
Bachelor’s degree and higher	3.11	1.74	3.17	4.71	3.72	3.48
Employed	44.06	45.65	41.30	38.82	47.69	47.66
Unemployed	28.99	23.56	30.82	30.59	29.21	32.95
Not in employment age	26.95	30.80	27.88	30.59	23.10	19.39
No disability	74.95	75.53	75.63	77.65	73.45	72.79
Some form of disability	25.05	24.47	24.37	22.35	26.55	27.21
Western Region	47.09	29.06	58.60	83.53	37.97	43.65
Central Region	29.17	26.03	26.08	7.06	36.80	27.67
Eastern Region	23.74	44.91	15.32	9.41	25.23	28.68
Quintile 1 (poorest)	14.03	24.11	10.20	2.35	14.66	12.97
Quintile 2	16.65	23.74	14.03	14.12	16.73	18.98
Quintile 3	18.90	20.81	18.12	21.18	18.91	21.12
Quintile 4	23.31	18.79	24.64	22.35	23.90	21.32
Quintile 5 (richest)	27.12	12.56	33.00	40.00	25.81	25.60
Health facilities within 1 h	87.09	82.95	90.61	94.12	83.70	83.56
Health facilities at more than 1 h	12.91	17.05	9.39	5.88	1630	16.44
Household size 3 members and below	27.22	25.94	28.57	16.47	26.34	24.20
Household size 4–5 members	42.49	39.96	43.48	42.35	42.43	43.85
Household size 6 members and above	30.29	34.10	27.95	41.18	31.23	31.95

Western region comprises of Thimphu, Chukha, Samtse, Paro and Haa; Central region comprises of WangduePhodrang, Punakha, Dagana, Tsirang, Gasa, Sarpang Bumthang, Zhemgang, Trongsa; Eastern region comprises of SamdrupJongkhar, Mongar, Trashigang, TrashiYangtse, PemaGatshel and Lhuntse

^a^Responses unavailable or respondents availed informal practitioners or private facilities for which categorization could not be established

Overall, 68.5% of those who were ill or injured in the last month used health care services in the month preceding the survey. For outpatient care, hospitals were used more frequently than primary health centers, while the usage of primary health centers was more prominent in rural areas. A higher proportion of females tend to use all categories of health services when ill or injured. The sample also displays regional variations in the use of health services, with the western region dominating the usage of all categories of health services. Except for primary health centers, usage of all other health services is progressively higher along the income gradient.

### Socio-economic determinants of health services utilization

Table 2 presents the logit coefficients for factors associated with the utilization of health services. Overall, there is 26% increase in the odds of utilizing outpatient health services when ill or injured in rural areas compared to urban areas. However, the odds of utilizing primary health care facilities compared to hospitals for outpatient services are 3.4 times higher in rural areas compared to urban areas. There is an insignificant difference in the utilization of inpatient hospital care between rural and urban areas. Women had 20% and 14% higher odds of consuming outpatient and inpatient health care services than their male counterparts. Consumption of outpatient care increased with age, while consumption of inpatient care decreased with age, consistently across urban and rural areas. Disability led to 2.3 times and 3.1 times higher odds of utilizing inpatient care in rural and urban areas, respectively.

**Table 2:** Factors associated with utilization of health services (logistic regression)

	**Total**			**Rural**			**Urban**		
	Used outpatient care in case of ill health or injury in the past month 1 = yes 0 = no	Type of outpatient care used when experienced ill health or injury in the past month 1 = primary health care 0 = outpatient hospital care	Used inpatient hospital care in the past year 1 = yes 0 = no	Used outpatient care in case of ill health or injury in the past month 1 = yes 0 = no	Type of outpatient care used when experienced ill health or injury in the past month 1 = primary health care 0 = outpatient hospital care	Used inpatient hospital care in the past year 0 = no 1 = yes	Used outpatient care in case of ill health or injury in the past month 1 = yes 0 = no	Type of outpatient care used when experienced ill health or injury in the past month 1 = primary health care 0 = outpatient hospital care	Used inpatient hospital care in the past year 1 = yes 0 = no
Area: (Ref: urban)									
Rural	1.26 *** (0.09)	3.40*** (0.36)	0.92 (0.06)						
Gender (Ref: male)									
Female	1.20*** (0.07)	1.22** (0.10)	1.14** (0.07)	1.16* (0.09)	1.30*** (0.13)	1.11 (0.08)	1.26** (0.12)	1.01 (0.17)	1.18 (0.11)
Age categories (Ref: age less than 30 years)									
Age 31–60 years	1.20* (0.11)	1.10 (0.16)	0.63*** (0.05)	1.29* (0.18)	0.98 (0.17)	0.55*** (0.06)	1.12 (0.15)	1.44 (0.39)	0.75** (0.09)
Age above 60 years	1.40*** (0.17)	0.99 (0.17)	0.67*** (0.07)	1.36* (0.22)	0.89 (0.18)	0.65*** (0.08)	1.68** (0.35)	1.37 (0.52)	0.66** (0.13)
Disability status (Ref: no disability)									
Some form of disability	0.90 (0.06)	0.84* (0.09)	2.55*** (0.17)	0.84* (0.08)	0.90 (0.10)	2.29*** 0.20)	1.00 (0.12)	0.68* (0.10)	3.13*** (0.34)
Marital status (Ref: never married)									
Married or together	1.21* (0.13)	1.16 (0.20)	2.36*** (0.23)	1.32* (0.23)	1.32 (0.28)	2.06*** (0.26)	1.11 (0.17)	0.85 (0.26)	2.73*** (0.40)
Divorced or separated	0.95 (0.14)	1.23 (0.27)	2.46*** (0.33)	1.11 (0.21)	1.33 (0.34)	2.20*** (0.37)	0.73 (0.17)	1.28 (0.57)	2.73*** (0.64)
Education (Ref: no formal education)									
Primary, secondary and vocational	0.94 (0.06)	0.83 (0.08)	0.70*** (0.05)	0.94 (0.09)	0.75** (0.09)	0.67*** (0.06)	0.95 (0.09)	0.96 (0.17)	0.74*** (0.07)
Bachelors degree and higher	0.75* (0.13)	1.36 (0.39)	0.53*** (0.08)	0.91 (0.31)	1.47 (0.62)	0.33*** (0.11)	0.76 (0.15)	1.16 (0.48)	0.67** (0.12)
Employment (Ref: employed)									
Unemployed	1.09 (0.08)	0.96 (0.10)	1.19** (0.08)	1.10 (0.11)	0.92 (0.11)	1.11 (0.10)	1.09 (0.13)	0.95 (0.21)	1.29** (0.14)
Not in employment age	1.83*** (0.20)	2.04*** (0.33)	1.09 (0.11)	1.83*** (0.28)	2.46*** (0.49)	0.87 (0.12)	1.86*** (0.29)	1.43 (0.41)	1.46** (0.24)
Geographical region (Ref: western region)									
Central region	0.47** (0.04)	1.00 (0.10)	0.93 (0.06)	0.44*** (0.04)	0.93 (0.11)	0.90 (0.08)	0.49*** (0.06)	0.98 (0.25)	0.98 (0.11)
Eastern region	0.63*** (0.05)	2.92*** (0.30)	1.28*** (0.09)	0.59*** (0.06)	2.36*** (0.28)	1.34*** (0.12)	0.68*** (0.09)	5.30*** (1.01)	1.12 (0.14)
Household consumption quintiles (Ref: quintile 1, poorest)									
Quintile 2	1.07 (0.11)	0.77** (0.10)	1.34*** (0.13)	1.12 (0.13)	0.73** (0.10)	1.45*** (0.15)	0.76 (0.21)	1.31 (0.58)	0.84 (0.21)
Quintile 3	1.02 (0.11)	0.67*** (0.09)	1.44*** (0.14)	0.97 (0.11)	0.66*** (0.09)	1.50*** (0.17)	0.91 (0.24)	0.89 (0.38)	1.02 (0.23)
Quintile 4	0.92 (0.10)	0.58*** (0.08)	1.36*** (0.14)	0.96 (0.12)	0.48*** (0.07)	1.58*** (0.19)	0.72 (0.18)	1.10 (0.44)	0.83 (0.19)
Quintile 5, richest	0.94 (0.14)	0.32*** (0.05)	1.49*** (0.16)	1.00*** (0.14)	0.31*** (0.05)	1.66*** (0.21)	0.73 (0.18)	0.48* (0.20)	0.94 (0.21)
Distance to health facilities (Ref: health facilities within 1 h)									
Health facilities at more than 1 h	0.65*** (0.06)	0.96 (0.11)	1.07 (0.08)	0.65*** (0.06)	0.97 (0.11)	1.04 (0.08)	0.55 (0.22)	0.57 (0.61)	2.06** (0.66)
Household size (Ref: household size of 3 members and below)									
Household size of 4–5 members	0.90 (0.07)	1.22* (0.13)	1.03 (0.07)	0.93 (0.09)	1.20 (0.15)	0.99 (0.09)	0.87 (0.09)	1.23 (0.24)	1.06 (0.11)
Household size of 6 members and above	0.87* (0.07)	1.40*** (0.16)	0.89 (0.07)	0.89 (0.09)	1.37** (0.17)	0.82** (0.08)	0.88 (0.11)	1.38 (0.32)	1.00 (0.13)
Constant	2.0*** (0.30)	0.12*** (0.03)	0.02*** (0.002)	2.41*** (0.49)	0.45*** (0.12)	0.02*** (0.003)	2.52*** (0.71)	0.09*** (0.04)	0.02*** (0.004)
Number of observations^(a)^	5904	4021	48639	3304	2210	29085	2600	1811	19554
Pseudo r-squared	0.0262	0.1641	0.0389	0.0283	0.0801	0.0391	0.0264	0.0879	0.0448

Standard errors in parentheses

For outpatient care, the sub-sample is extracted conditional on reporting ill/injured in the last one month. For inpatient care, full sample is used to examine utilization pattern in the last one year. Further explanation is provided in the methods section

^*^ p < 0.1

^**^ *p* < 0.05

^***^ *p* < 0.01

Residence in a particular geographical region was also found to play a significant role in utilizing outpatient health services. Utilization reduced as we move away from the western region, consistently for both urban and rural areas. Eastern region recorded 2.9 times higher odds of encountering primary health care services than hospital services.

We observe progressively increasing odds of utilization of inpatient services for the higher-income quintile in rural areas, while we do not observe meaningful differences between income quintiles in urban areas. Usage of primary health care services declined among the population in richer quintiles in rural areas. Larger distance to health facilities led to 35% reduction in the odds of utilizing outpatient health services in rural areas and more than two-fold higher odds of utilizing inpatient services in urban areas.

### Decomposition of rural–urban differences in utilization of health services

Table 3 shows the decomposition of differences in utilization of health services between urban and rural areas. The results indicate that there is a probability difference of 0.03 in the utilization of outpatient care conditional on ill health and injury in the past month between urban and rural areas. The probability difference changes to 0.29 between urban and rural areas when the type of outpatient care used is a primary health center rather than a hospital. For the use of inpatient care in the preceding year, the corresponding probability difference is 0.001. The difference in probability of health care use is overwhelmingly driven by endowment effects (254.3% for outpatient and 263.1% for inpatient care), denoting a strong presence of characteristics difference among these variables and offsetting a significant coefficient effect in the opposite direction. In other words, the analysis separates the contributions of the factors into endowment and coefficient effects, arithmetically denoted as positives and negatives, and therefore, it is possible to have higher than 100% in either direction, which offsets positive and negative effects against each other. For usage of primary health care centers rather than hospitals, 36% of the difference is attributed to endowment effects, allowing for a major presence of unexplained differences or different treatments on the same characteristics.

**Table 3:** Decomposition analysis (Fairlie) showing the percentage contribution of each covariate to the gaps in utilization of health services between urban and rural areas

	**Used outpatient care in case of ill health or injury in the past month** **1 = yes** **0 = no**			**Type of outpatient care used when experienced ill health or injury in the past month** **1 = primary health care** **0 = outpatient hospital care**			**Used inpatient hospital care in the past year** **1 = yes** **0 = no**		
Pr Urban	0.6965			0.1121			0.0301		
Pr Rural	0.6689			0.4018			0.0312		
Difference	0.0277			-0.2897			-0.0011		
Endowment effect (%)	0.0817 (254.3%)			-0.1042 (36.0%)			-0.0028 (263.1%)		
**Detailed decomposition of endowment effect**	**Coef**	**SE**	**%**	**Coef**	**SE**	**%**	**Coef**	**SE**	**%**
Female	-0.0009	0.0005	-0.9%	-0.0012***	0.0006	1.2%	0.0001	0.0001	-4.7%
Age 31–60 years	-0.0016	0.0020	-1.6%	0.0004	0.0032	-0.4%	0.0006	0.0005	-30.4%
Age above 60 years	-0.0129**	0.0051	-12.4%	0.0028	0.0047	-2.7%	0.0015***	0.0005	-79.7%
With disability	-0.0001	0.0027	-0.1%	0.0021	0.0022	-2.0%	-0.0023***	0.0004	120.2%
Quintile 2	0.0084	0.0087	8.1%	0.0098**	0.0041	-9.4%	-0.0017***	0.0006	90.4%
Quintile 3	0.0012	0.0033	1.1%	0.0032**	0.0013	-3.0%	-0.0006***	0.0003	29.7%
Quintile 4	-0.0066	0.0050	-6.3%	-0.0179***	0.0040	17.2%	0.0014***	0.0005	-70.8%
Quintile 5	-0.0189	0.0148	-18.2%	-0.0649***	0.0090	62.3%	0.0041***	0.0012	-211.2%
Education: Primary, secondary and vocational	-0.0020	0.0042	-1.9%	-0.0111**	0.0044	10.6%	-0.0025***	0.0006	131.3%
Education: Bachelors and higher	-0.0026	0.0019	-2.5%	0.0027	0.0030	-2.6%	-0.0014***	0.0003	70.2%
Married/together	-0.0019	0.0028	-1.9%	-0.0063	0.0050	6.1%	-0.0003	0.0007	17.2%
Divorced/separated	0.0037	0.0028	3.6%	-0.0031	0.0028	3.0%	-0.0012***	0.0005	60.0%
Unemployed	0.0017	0.0024	1.7%	-0.0013	0.0018	1.2%	0.0004	0.0003	-19.4%
Not in employment age	0.0134***	0.0039	12.9%	0.0264***	0.0057	-25.3%	-0.0002	0.0003	9.7%
Central region	0.0418***	0.0073	40.3%	0.0035	0.0054	-3.4%	0.0006	0.0005	-32.6%
Eastern region	0.0181***	0.0067	17.5%	-0.0468***	0.0065	44.9%	-0.0018***	0.0006	93.7%
Health facilities at more than 1 h	0.0289	0.0201	27.9%	0.0014	0.0045	-1.3%	-0.0003	0.0006	16.3%
Household size of 4–5 members	-0.0029	0.0024	-2.8%	0.0036	0.0025	-3.4%	-0.00004	0.0003	1.9%
Household size of 6 members and above	0.0035	0.0036	3.3%	-0.0074**	0.0031	7.1%	0.0009	0.0005	-46.8%

^*^ *p* < 0.1

^**^ *p* < 0.05

^***^ *p* < 0.01

**Fig. 1:**
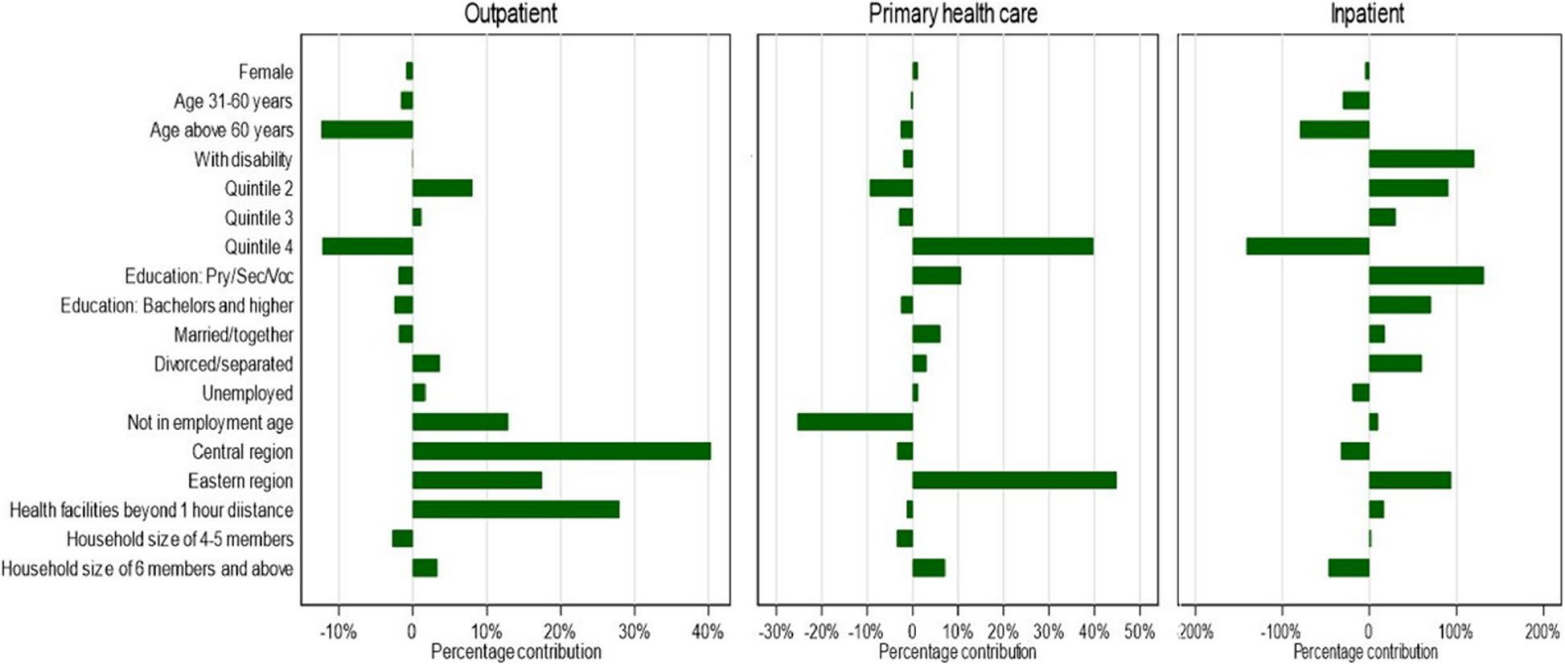
Fairlie decomposition of difference in utilization (endowment effects) between urban and rural areas

Among the endowment component, the geographical region contributed to widening the difference while increasing age contributed to reducing the differences in utilization of outpatient services. The primary contributors to differences in using primary health care centers rather than hospitals for outpatient care were higher income levels and living in the eastern region. For inpatient services, the top three contributors to differences in utilization of inpatient services were education, disability status and living in the eastern region. We visually illustrate the decomposition results estimating the percentage contribution to the observed differences for the explained component in Fig. 1. A positive contribution of a covariate indicates widening the utilization gap between urban and rural populations, while the negative contribution of a covariate indicates its contribution in reducing the gap.

### Decomposition of the income-related inequality in utilization of health services

**Fig. 2:**
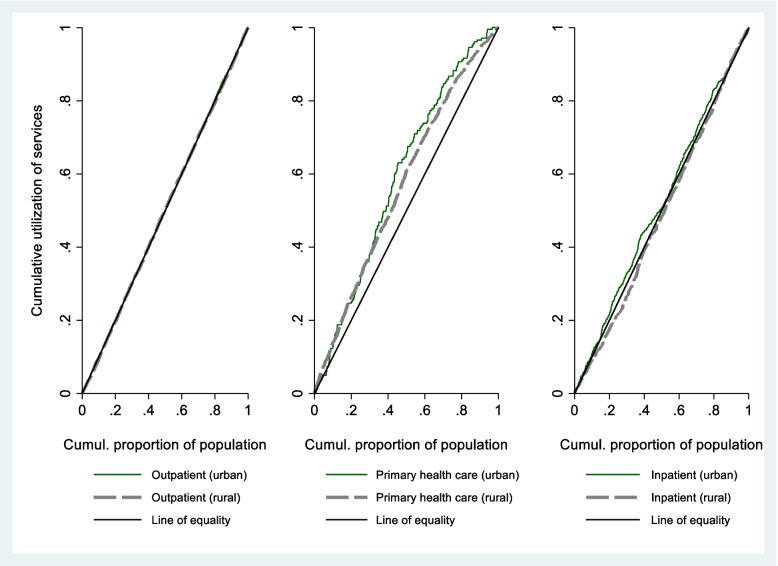
Concentration curves for utilization of health services

The concentration indices for contacts with and the type of facilities used for outpatient care as well as the utilization of inpatient care are presented in Table 4, together and disaggregated by rural and urban areas. The corresponding concentration curves are presented in Fig. 2. We observe a significant pro-poor tendency for contacts with primary health centers instead of hospitals, pointing toward higher usage of primary health care among individuals belonging to lower-income households, consistently across, though more prominently in rural areas than in urban areas. We do not find any significant evidence to suggest income-related inequality in total outpatient contacts and use of inpatient health care services, overall as well as separately for rural and urban areas.

**Table 4:** Concentration indices

**Utilization**	**Observations**	**Total**		**Urban**		**Rural**	
		**CI**	***P* value**	**CI**	***P* value**	**CI**	***P* value**
Used outpatient care in case of ill health or injury in the past month 0 = no 1 = yes	Total: 5904 Urban = 2600 Rural = 3304	0.0161	0.0221	0.0043	0.6770	0.0146	0.1224
Type of outpatient care used when experienced ill health or injury in the past month 1 = primary health care 0 = outpatient hospital care	Total: 4021 Urban = 1811 Rural = 2210	-0.1550	0.0000	-0.0498	0.0000	-0.1230	0.0000
Used inpatient hospital care in the past year 0 = no 1 = yes	Total: 48639 Urban = 19554 Rural = 29085	0.0011	0.2241	-0.0009	0.5385	0.0038	0.0007

*CI* Concentration index

Table 5 shows the decomposition of Erreygers’ corrected concentration indices for contacts with and type of facilities used for outpatient care as well as utilization of inpatient care. The results are presented as aggregate decomposition (the contribution of changes in the entire set of explanatory variables), and detailed decomposition (identifying the individual contribution of each determinant to the observed difference). The endowment effect reflects the contribution of each determinant to the difference in utilization gap between rural and urban areas, while the coefficient effect indicates the discrimination or unequal treatment of the rural against the urban.

**Table 5:** RIF decomposition of Erreygers’ concentration indices for inequality in utilization of health services between urban and rural areas

	**Used outpatient care in case of ill health or injury in the past month** **1 = yes** **0 = no**				**Type of outpatient care used when experienced ill health or injury in the past month** **1 = primary health care** **0 = outpatient hospital care**				**Used inpatient hospital care in the past year** **1 = yes** **0 = no**			
**Aggregate decomposition**	**Coefficient (SE)**				**Coefficient (SE)**				**Coefficient (SE)**			
group_1 Urban	-0.0006 (0.0096)				-0.0351*** (0.0076)				-0.0012 (0.0012)			
group_2 Rural	0.0011 (0.0093)				-0.1002*** (0.0111)				0.0012 (0.0013)			
Difference	-0.0017 (0.0145)				0.0652*** (0.0140)				-0.0024 (0.0018)			
Endowment effect	0.0342* (0.0174)				-0.0436** (0.0172)				-0.0031 (0.0019)			
Coefficient effect	-0.0358 (0.0219)				0.1088*** (0.0215)				0.0007 (0.0028)			
**Detailed decomposition**	**Endowment effect**	**SE**	**Coefficient effect**	**SE**	**Endowment effect**	SE	**Coefficient effect**	**SE**	**Endowment effect**	**SE**	**Coefficient effect**	**SE**
Female	-0.0001	0.0005	-0.0008	0.0175	0.0005	0.0007	0.0037	0.0168	0.00001	0.00002	-0.0012	0.0019
Age 31–60 years	-0.0013	0.0025	0.0083	0.0183	-0.0023	0.0036	-0.0038	0.0157	0.0002**	0.0001	0.0004	0.0019
Age above 60 years	-0.0074	0.0052	0.0003	0.0061	0.0017	0.0052	0.0055	0.0056	0.0007	0.0004	-0.0001	0.0006
With disability	0.0014	0.0026	0.0029	0.0077	0.0001	0.0028	-0.0011	0.0062	0.0004	0.0002	0.0015*	0.0008
Quintile 2	0.0011	0.0036	0.0082	0.0082	-0.0127**	0.0052	-0.0151*	0.0086	0.0012**	0.0005	0.0024*	0.0013
Quintile 3	-0.0003	0.0015	0.0049	0.0134	-0.0048**	0.0020	-0.0154	0.0130	0.0004**	0.0002	0.0033	0.0023
Quintile 4	0.0009	0.0031	0.0188	0.0235	0.0032	0.0039	-0.0147	0.0227	-0.0007*	0.0004	0.0068*	0.0036
Quintile 5	0.0098	0.0144	0.0188	0.0424	-0.0151	0.0138	0.0062	0.0349	-0.0021	0.0018	0.0117**	0.0055
Education: Pry/Sec/Voc	-0.0037	0.0039	-0.0048	0.0138	-0.0026	0.0048	-0.0070	0.0159	0.0003	0.0005	-0.0018	0.0028
Education: Bachelors and higher	0.0026	0.0034	-0.0060	0.0049	0.0047	0.0049	-0.0097	0.0067	-0.0010**	0.0005	0.0009	0.0008
Married/together	0.0040*	0.0035	-0.0107	0.0252	-0.0140**	0.0058	-0.0719**	0.0233	-0.0002	0.0001	-0.0037	0.0028
Divorced/separated	0.0049	0.0025	-0.0004	0.0046	-0.0010	0.0030	-0.0049	0.0040	-0.0003	0.0002	0.0002	0.0005
Unemployed	-0.0010	0.0022	-0.0021	0.0121	0.0050**	0.0023	-0.0240**	0.0121	0.0001	0.0003	0.0005	0.0019
Not in employment age	-0.0010	0.0047	0.0039	0.0162	0.0126*	0.0067	-0.0506**	0.0200	-0.0001	0.0001	-0.0006	0.0017
Central region	0.0265**	0.0063	0.0259	0.0060	-0.0152**	0.0067	-0.0046	0.0046	-0.0009	0.0006	0.0007	0.0009
Eastern region	-0.0038	0.0052	0.0062	0.0043	-0.0014	0.0069	-0.0082*	0.0047	-0.0008	0.0006	0.0005	0.0007
Health facilities beyond 1 h distance	-0.0019	0.0050	0.0007	0.0007	-0.0053	0.0049	-0.0005	0.0010	0.0000	0.0006	-0.0002	0.0002
Household size of 4–5 members	0.0042	0.0026	-0.0121	0.0163	-0.0046	0.0031	0.0189	0.0179	0.0010***	0.0004	-0.0056**	0.0024
Household size of 6 members and above	-0.0007	0.0033	0.0023	0.0096	0.0077**	0.0034	0.0212***	0.0081	-0.0013**	0.0005	-0.0039***	0.0013
Constant			-0.1004	0.0996			0.2848***	0.1002			-0.0112	0.0143

*SE* standard error

^*^ p < 0.1

^**^ *p* < 0.05

^***^ *p* < 0.01 using bootstrap SE using 100 reps

For utilization of outpatient services, living in the central region had a significant influence on income-related inequality*.* The variables influencing the usage of primary health centers for outpatient care were income, married/together status, unemployment, living in the central region and household size of more than six members. For inpatient care, income, age 31–60 years, education of bachelors level and higher and household size of four members and higher emerged as the significant drivers influencing the inequality gap.

## Discussion

Our study establishes that there are minimal differences in the utilization of health services among rural and urban areas in Bhutan, with a mildly higher utilization of outpatient care in urban areas (probability difference of 0.03) and almost similar rates in utilization of inpatient care between the two settings (probability difference of 0.001). However, there is a significant difference in the type of outpatient facility used when ill or injured, with more than three-fold probability of using primary health centers compared to outpatient hospital facilities in rural areas.

Our study generally conforms to the previous round of data and analysis in Bhutan, which revealed that living in rural areas with longer travel time led to a higher tendency to visit primary health care facilities and lesser propensity of getting care from secondary and tertiary providers [16]. Another study established that place of residence is significantly associated with choice of health facility [17]. It adds to the evidence base that while the differences are not so glaring in terms of average utilization of health services, there are significant differences in the level of services used among rural and urban settings. Given the considerable differences in health outcomes between rural and urban Bhutan [11, 13–15], this leads us to speculate that the differences in outcomes may be attributed to quality of services and inefficient referral system rather than the contacts with or utilization of these services. Women used more health care than men irrespective of a rural or urban setting. This is consistent with several studies in large number of settings which show that women use more health care services than men [40]. The utilization rate for women was higher than for men, even after discounting the usage of childbirth services, which merits further investigation to understand the disease patterns and health seeking behaviour.

The narrowing utilization rates between the rural and urban settings could have been a result of the expansion of primary health infrastructure in the last three decades. Bhutan went through a fierce expansionary policy from the 1980s to early 2010s, where the predominant health development approach was the expansion of health infrastructure facilities. Despite the difficult geographical terrain and dispersed population settlements, access to health services has remarkably improved with higher utilization of primary-level care and more rural residents expressing satisfaction with services [11]. From just 2 hospitals and 11 dispensaries in 1961, Bhutan had 51 hospitals and 238 primary health centers (including 53 sub-facilities) in 2021 [41]. While the huge expansion in the reach of primary health care is particularly noteworthy, the trend may have created imbalances in access to quality and higher levels of care in rural and remote locations, possibly due to the sub-optimal quality of care or absence of a robust referral mechanism. High incidence of bypassing primary health care to avail higher level of care in the hospitals have been documented [11, 16, 18], which indicates an interplay of supply and demand-driven factors, that merits comprehensive investigations. Considering Bhutan’s continuing shortage of health workers, particularly physicians and physician specialists [11] and the limited available budgetary space for health, future policies and strategies for expansion of health services to unreached areas, however, may necessitate a careful consideration of the economies of scale and scope.

The rural–urban gap in utilization of outpatient services is primarily driven by residence in a particular geographical region. Outpatient contacts reduced as we move away from the western region towards the central and eastern regions, consistently for both urban and rural areas. This conforms to the generally higher level of socio-economic status in the western region [10] and higher density of health facilities in the western region [11]. The high level of rural–urban differences in using primary health care compared to hospital outpatient services was primarily explained by income levels (4th and 5th quintiles) and living in the eastern region. For differences in inpatient care, the contributors were income, age above 60 years, education and marital status, in addition to living in the eastern region. Older age groups, particularly those over 60 years, contributed to reducing the differences in utilization of health services between rural and urban areas. The heterogeneity of drivers of utilization of outpatient and inpatient health services may have stemmed from the considerable geographical differences in supply-side characteristics such as density and levels of health facilities [11], the different nature of these services, the demand-side considerations (health-seeking behaviour) for these different types of services and their impact on service utilization.

We find mixed evidence for income-related inequalities in utilization of health services. We do not find any significant evidence to suggest income-related inequality in both total outpatient contacts and utilization of inpatient health care services, overall as well as independently in rural and urban areas. However, a pro-poor tendency was observed for utilization of primary health centers compared to hospitals for outpatient services, indicated by higher utilization of primary health centers among individuals belonging to lower-income households, implying higher consumption of hospital outpatient resources by richer households. The differences were consistently and more prominent in rural areas than in urban areas. This indicates that while inequality permeates the rural–urban characteristics and affects both areas, inequality is more severe in rural settings. Such inequalities were influenced by income, marital status, employment status, household size and residence in the central region. Largely conforming to the literature examined on health inequalities in countries [21–25], socio-economically vulnerable population groups are the worst hit.

Looking at factors contributing to the mean effect and distribution effect of decompositions, we observe similarities and differences in the significance and importance rankings of the explanatory variables. For example, the main factor contributing to the rural–urban gap in outpatient services was residence in a particular geographical region, similar to the significant factor that influenced income-related inequality. While higher income levels and living in the eastern region emerged as the primary drivers of the rural–urban gap in the utilization of primary health care services, income-related inequality was influenced by marital and unemployment status of household head besides residence in the central region, income and household size of more than six members. For inpatient care services, while the top three drivers of the rural–urban gap were income, residence in the eastern region, and household head’s age, education and marital characteristics, income-related inequality was influenced by household size and education level of household head, besides income and age. These findings establish that factors influencing income-related inequality do not necessarily follow those influencing the utilization gaps. A prominent illustration of this difference is that while utilization gaps were consistently more pronounced for people residing in the eastern region, inequalities in utilization were driven by residence in the central region of the country. Policies, which take cognizance of these differences, may stand better informed, targeted and potentially more effective for progress towards UHC.

This study bears several limitations. First, we may have missed out on other potential factors affecting the rural–urban differences in health care use and inequality due to the limitations of the survey data. For example, households with chronic care needs would influence the utilization of health services, thus resulting in health disparities. Such data were, however, not available in the dataset we used. Similarly, the use of health services abroad on one's own choice, a trend picking up quickly in Bhutan, is expected to have a slightly different utilization pattern and socio-economic profile, which our datasets do not separately capture. We ensured, however, that most determinants of health service use suggested in the literature have been incorporated into the study. Finally, in the absence of an objective measure of health status, we used self-reported health, a subjective measure that may suffer from potential response bias.

## Conclusion

Considering the significant differences in health outcomes between rural and urban areas of the country, we designed this study to measure the utilization pattern and its socio-economic determinants, and decompose the factors behind the differences. We find that significant inequalities persist in the level of services used for outpatient care with more than three-fold probability of using primary health centers compared to hospitals in rural areas compared to urban areas. We also find that the use of primary health care is pro-poor and that hospital resources are concentrated among the wealthier segment of the population. While inequality permeates the rural–urban characteristics and affects both areas, inequality is more severe in rural settings. However, we do not find significant evidence of utilization differences or inequality in the use of inpatient health care services.

This study provides helpful evidence for policy reassessment to address health access and equality challenges in Bhutan. First, as discussed, supply-side factors are the key to addressing socio-economic-related health inequalities for progress towards UHC in Bhutan. This would entail taking more quality, secondary and tertiary level health care services closer to people in rural areas through policies that promote access to higher levels and quality of health services. Considering the immediate limitations in resources, it would entail more strategic longer-term planning as well as health facilities reorganization and the use of digital or e-health tools, among others, in the short and medium terms. Second, this study reconfirms the success of primary health care in Bhutan and recommends that its prominence and strength in the health system organization should continue. However, considering the continuing differences in critical health outcomes between rural and urban areas of the country, the study suggests a reassessment of the quality of primary health care, particularly in terms of scope of services provided, supply-side readiness and patient referral framework. Third, as evident from this study, income, disability status, marital status, education level, household size and employment status are critical drivers of health inequality that should be featured in multi-sectoral discussions and policy processes. The health agenda needs to feature more prominently in discussions around socio-economic and social protection policies.

We also demonstrate that, while there are obvious overlaps, factors influencing income-related inequality are not necessarily the same as those influencing the utilization gaps. Future health equity research and policies in low- and middle-income countries, which take cognizance of these differences, may stand better informed, targeted and potentially more effective.

## Data Availability

The datasets used and/or analysed during the current study are available from the corresponding author on reasonable request and with permission of National Statistics Bureau.

## Footnotes

1 Based on annual average for 2017 calculated by the Royal Monetary Authority of Bhutan (1 USD = 66.43 Ngultrum).
